# Long-term mechanical loading aggravates osteoarthritis through a pro-apoptotic inflammatory microenvironment

**DOI:** 10.7555/JBR.39.20250025

**Published:** 2026-03-19

**Authors:** Shiyun Shen, Tianshun Fang, Jiabao Dong, Junjie Li, Miyang Xu, Jian Wu, Jiangfeng Zhang, Jianyou Li, Wei Liu, Wei Zhou, Xiongfeng Li

**Affiliations:** 1 Huzhou Central Hospital, Fifth School of Clinical Medicine of Zhejiang Chinese Medical University, Huzhou, Zhejiang 313000, China; 2 Huzhou Central Hospital, Affiliated Central Hospital of Huzhou University, Huzhou, Zhejiang 313000, China; 3 Department of Urology, Renji Hospital Affiliated, Shanghai Jiao Tong University School of Medicine, Shanghai 200127, China

**Keywords:** osteoarthritis, mechanical loading

## Abstract

Articular cartilage maintains joint homeostasis by adapting to mechanical loading, but both insufficient and excessive loading can impair cartilage integrity. Whether mechanical activity should be restricted in early osteoarthritis (OA), particularly among exercise enthusiasts, remains controversial. Here, we established *in vitro* and *in vivo* models of prolonged moderate mechanical loading (7.5% strain, 1 Hz) and analyzed human cartilage from weight-bearing and non-weight-bearing regions using RNA sequencing. Prolonged exposure (≥ 12 h) significantly increased chondrocyte apoptosis (2.3-fold), reduced expression of the chondrogenic transcription factor SOX9 and the matrix markers COL2A1, and elevated nerve growth factor (NGF) expression (1.8-fold), accompanied by enrichment of neural sensitization and inflammatory pathways. Immunofluorescence staining revealed NGF accumulation in mechanically stressed cartilage. Unlike high-intensity stress, which led to immediate apoptosis, moderate loading induced a delayed pro-apoptotic response after 12 h. These findings indicate that prolonged moderate mechanical loading may promote chondrocyte apoptosis through an NGF-mediated inflammatory microenvironment and provide mechanistic evidence suggesting that patients with early OA may benefit from limiting high-impact or prolonged moderate-intensity exercise sessions to prevent cartilage damage and guide rehabilitation.

## Introduction

Osteoarthritis (OA), characterized by joint pain, swelling, and mobility issues, accounts for approximately 50% of all chronic pain cases among individuals aged 65 and above, primarily stemming from cartilage degeneration^[1]^. According to statistics from the World Health Organization (WHO), the global number of individuals with symptomatic OA has exceeded 528 million^[2]^. The pathological manifestations of OA include the degradation of articular cartilage, subchondral osteosclerosis, and the formation of osteophytes^[3]^. Currently, a stepped treatment approach is employed in clinical practice, which includes exercise guidance, physiotherapy, pharmacotherapy, and joint replacement surgery^[4–5]^. Exercise rehabilitation is a fundamental component of these comprehensive treatment regimens, encompassing localized muscle-strengthening exercises as well as systemic aerobic exercises^[6]^.

Exercise therapy demonstrates varying applicability across different stages of OA. While moderate exercise is widely acknowledged to alleviate OA symptoms, the extent to which long-term adherence to exercise habits can improve knee cartilage remains a subject of controversy, particularly for individuals who have already developed OA symptoms. This complexity arises from the dual nature of mechanical loading, which does not consistently exert a positive influence on the progression of OA^[7]^. Moderate mechanical loading has been demonstrated to decrease the expression of proteolytic enzymes and impede the progression of OA^[6,8–9]^. In contrast, microgravity conditions in the space environment can damage cartilage^[10]^. Animal studies have revealed that maintaining normal mechanical joint loading through moderate physical activity, as opposed to remaining sedentary, can protect against cartilage degradation in rats^[11]^. Excessive mechanical loading, however, results in various detrimental effects on articular cartilage, including edema, fissures, fibrillation, local erosion, fragmentation, and osteophyte formation^[12–13]^. Weight loss demonstrates a dose-response relationship with symptomatic improvement because of the reduction of mechanical loading on the musculoskeletal system^[14]^. In the microenvironment of articular cartilage, excessive mechanical loading triggers the expression of inflammatory factors such as interleukin-1β (IL-1β), interleukin-6 (IL-6), prostaglandin E2 (PGE2), tumor necrosis factor-α (TNF-α), and cyclooxygenase-2 (COX-2)^[15–16]^. The development of OA is not only influenced by biomechanical factors but is also closely associated with the complex interplay between inflammation and pain. While mechanical loading directly impacts cartilage integrity, it also modulates the joint microenvironment, including nerve-related pathways. Pain, a hallmark symptom of OA, is increasingly recognized as a product of neuroinflammatory processes involving nerve sensitization and signaling molecules such as nerve growth factor (NGF). In recent years, NGF has also emerged as a therapeutic target in OA management. Several clinical trials have evaluated NGF-neutralizing antibodies such as tanezumab, which have demonstrated significant efficacy in reducing pain and improving function in patients with moderate-to-severe OA. However, these treatments have also been associated with risks, including the development of rapidly progressive OA, particularly when combined with nonsteroidal anti-inflammatory drugs. This paradox highlights the complex role of NGF—not only as a driver of nociceptive pain but also as a regulator of joint homeostasis—making it a "double-edged sword" in OA therapy. As such, NGF is increasingly recognized as both a biomarker and a therapeutic target in OA, and its regulation must be contextualized within biomechanical loading conditions^[17–19]^.

Despite increasing attention to NGF-targeted therapies, the upstream regulatory mechanisms of NGF expression in OA remain poorly understood. Notably, it is unclear how chronic, moderate mechanical loading—a common scenario in daily life and exercise—affects NGF signaling in articular cartilage. Previous studies have largely focused on acute or high-intensity mechanical insults, with limited exploration of long-term mechanical stimulation and its neuroinflammatory consequences^[20]^. The dual effect of exercise on chondrocytes has introduced uncertainty, as there remains ongoing debate about whether individuals with early-stage OA should reduce work and exercise intensity, particularly among athletic enthusiasts.

In the current study, we investigated how long-term mechanical loading influences the progression of OA. We employed both *in vivo* treadmill-based loading models and *in vitro* dynamic compression systems, supplemented by RNA sequencing of human cartilage.

## Materials and methods

### Chemicals and reagents

High-glucose DMEM and trypsin (Gibco, Grand Island, NY, USA), collagenase type Ⅱ (Gibco), fetal bovine serum (FBS; Hangzhou Sijiqing Company, China), polyclonal rabbit anti-collagen Ⅱ antibody (1∶200; Cat. #ab34712, Abcam, Cambridge, UK), a reverse transcription kit (QIAGEN, Germany), Trizol reagent (Gibco), dimethyl sulfoxide (Sigma, St. Louis, MO, USA), six-well culture plates (Corning, NY, USA), pentobarbital sodium (Shanghai Chemical Reagent, Shanghai, China), and RPMI 1640 medium (Thermo Fisher Scientific, Waltham, MA, USA).

### Cell culture and mechanical loading

Cartilage was isolated from five 1-week-old Sprague-Dawley rats. The cartilage was diced into small pieces and digested with 0.2% collagenase Ⅱ at 37 ℃ for 4 h. Chondrocytes were cultured in DMEM supplemented with 10% FBS. Third-passage chondrocytes were seeded into six-well culture plates for cell culture using a tension plus system (Flexcell FX-4000; Flexcell International Corp., Hillsborough, NC, USA). Cells were divided into five groups and subjected to cyclic tensile strain (7.5%, 1 Hz) for 0 h (control), 3 h, 6 h, 9 h, or 12 h using the mechanical loading system, followed by an additional 12 h of incubation under static conditions before proceeding to subsequent experiments.

### Cell counting kit-8 (CCK-8) assay

Cell viability was evaluated using the CCK-8 assay kit (Dojindo, Kumamoto, Japan) according to the manufacturer's instructions. Briefly, CCK-8 solution was added to each well and incubated for 2 h, after which the absorbance at 450 nm was measured using a microplate reader to assess cell activity.

### Small interfering RNA (siRNA) transfection

siRNA targeting *Sox9* (si-*Sox9*) and a non-targeting control (si-NC) (GenePharma, Shanghai, China) were transfected into third-passage chondrocytes at a confluence of 60%–70% using Lipofectamine 3000 (Invitrogen, Carlsbad, CA, USA) in antibiotic-free Opti-MEM medium following the manufacturer's instructions (final siRNA concentration: 50 nmol/L). After 6 h, the medium was replaced with DMEM supplemented with 10% FBS, and cells were cultured for 48 h before mechanical loading or RNA extraction. Knockdown was verified by reverse transcription-quantitative PCR (RT-qPCR; ≥ 70% reduction in *Sox9* mRNA levels).

### Bulk RNA sequencing (RNA-seq)

Articular cartilage samples were collected from the weight-bearing and non-weight-bearing zones of the same patients with end-stage OA during total joint arthroplasty. Tissues were harvested in TRIzol reagent (Cat. #9109, Takara, Kyoto, Japan) for RNA extraction. Human cartilage samples were obtained with written informed consent from all donors. All experimental procedures involving human tissues were approved by the Institutional Review Board of Huzhou Central Hospital (Approval No. 202203002) and conducted in accordance with the Declaration of Helsinki. Experienced orthopedic surgeons harvested cartilage from the weight-bearing and non-weight-bearing zones within the same joint, as confirmed by imaging and intraoperative assessment. After ensuring the quality of RNA, samples were reverse transcribed, and cDNA libraries (Biozeron, Shanghai, China) were constructed for sequencing. Differential expression analysis was performed using DESeq2 (R v4.2.2), with differentially expressed genes defined as |log_2_(fold change)| > 1.5 and adjusted *P*-values (false discovery rate [FDR]) < 0.05 based on the Benjamini–Hochberg correction. Gene Ontology (GO) enrichment and Gene Set Enrichment Analysis (GSEA) were conducted using the clusterProfiler package in R, with the normalized enrichment score (NES) and FDR values reported. Heatmaps and volcano plots were generated using the pheatmap and ggplot2 packages.

### RT-qPCR analysis

Total RNA was isolated from cells using TRIzol reagent (Cat. #9109, Takara) according to the manufacturer's instructions. RNA concentration and purity were assessed using a NanoDrop spectrophotometer. Subsequently, 1 μg of RNA was reverse-transcribed into cDNA using a PrimeScript RT reagent kit (Takara). RT-qPCR was conducted using SYBR Green Master Mix on a LightCycler 480 Ⅱ System (Roche Diagnostics, Basel, Switzerland). Relative gene expression levels were calculated using the 2^−ΔΔCt^ method, with *Actb* used as the internal control. The primer sequences used for RT-qPCR are listed in ***Table 1***.

**Table 1:** Sequences of primers used for reverse transcription-quantitative PCR

Rat genes	Direction	Sequence (5′-3′)
*Sox9*	Forward	CCAGAGAACGCACATCAAGACG
	Reverse	TGTAGGTGAAGGTGGAGTAGAGCC
*MMP9*	Forward	CTTGAAGTCTCAGAAGGTGGATC
	Reverse	CGCCAGAAGTATTTGTCATGG
*Col2a1*	Forward	TGCTGGAAAACCTGGTGATGATG
	Reverse	TAACCTCTGTGACCCTTGACAC
*Acan*	Forward	TGTGTCAGTGGTGCCCTCTC
	Reverse	GGCTCCCATTCAGTCTGTTTTTC
*Actb*	Forward	CACGATGGAGGGGCCGGACTCATC
	Reverse	TAAAGACCTCTATGCCAACACAGT

### Immunofluorescence (IF) staining

Cells were fixed with 4% paraformaldehyde (PFA) for 15 min at room temperature and then permeabilized using 0.2% Triton X-100 for 15 min. After washing with phosphate-buffered saline (PBS), cells were blocked using 5% bovine serum albumin for 1 h at 37 ℃, and incubated with the primary antibody at 4 ℃ overnight. They were then incubated with the secondary antibody for 2 h and DAPI for 15 min at 37 ℃. Images were captured using a fluorescence microscope (Olympus BX61, Tokyo, Japan). Primary antibodies and dilutions were as follows: anti-SOX9 (1∶200; Cat. #ab185230, Abcam), anti-ACAN (1∶200; Cat. #13880-1-AP, Proteintech, Chicago, IL, USA), anti-IL-1β (1∶200; Cat. #ab9722; Abcam), and anti-NGF (1∶200; Cat. #ab52918; Abcam).

### Flow cytometry analysis

Mechanically loaded and control rat chondrocytes were fixed using 1% (w/v) PFA. Approximately 5 × 10^5^ cells were then incubated on ice with 1 μg APC- or PE-conjugated antibody for 30 min. Cell apoptosis was assessed using an Annexin Ⅴ-FITC/PI apoptosis detection kit (Cat. #BL107A, BioSharp, Hefei, China). After washing with PBS, the samples were analyzed by flow cytometry (Beckman Coulter, Brea, CA, USA).

### Animal models

To evaluate the effects of long-term mechanical loading on cartilage in the early stages of OA, we performed destabilization of the medial meniscus (DMM) modeling in C57BL/6 mice (*n* = 5 for each group), which were obtained from the Laboratory Animal Center of Hangzhou Medical College (Hangzhou, China). The research scheme was approved by the Institutional Animal Care and Use Committee of Huzhou Central Hospital (Approval No. 202305003). All applicable institutional and national guidelines were followed. Mice were anesthetized with pentobarbital sodium (100 μL/10 g, 0.8%, i.p.). After anesthesia, an incision was made in the knee joint of the mice, and the patellar tendon was gently displaced to fully expose the knee joint capsule. The medial meniscotibial ligament anchoring the medial meniscus to the tibia was sharply transected using microsurgical scissors. The five mice in the experimental group underwent treadmill training (running for 0.5 h, five times a week) following DMM modeling to apply mechanical loading. Mice were euthanized by cervical dislocation under deep anesthesia.

### Statistical analysis

All data were presented as mean ± standard deviation and analyzed using both SPSS 22.0 and GraphPad Prism (v8.0.1). Depending on data distribution and sample size, comparisons between two groups were performed using an unpaired Student's *t*-test, where comparisons among multiple groups were conducted using one-way ANOVA or Kruskal–Wallis test. Bonferroni correction was applied when multiple comparisons were involved. A *P* < 0.05 was considered statistically significant.

## Results

### The apoptosis of chondrocytes was aggravated by long-term mechanical loading

**Figure 1:**
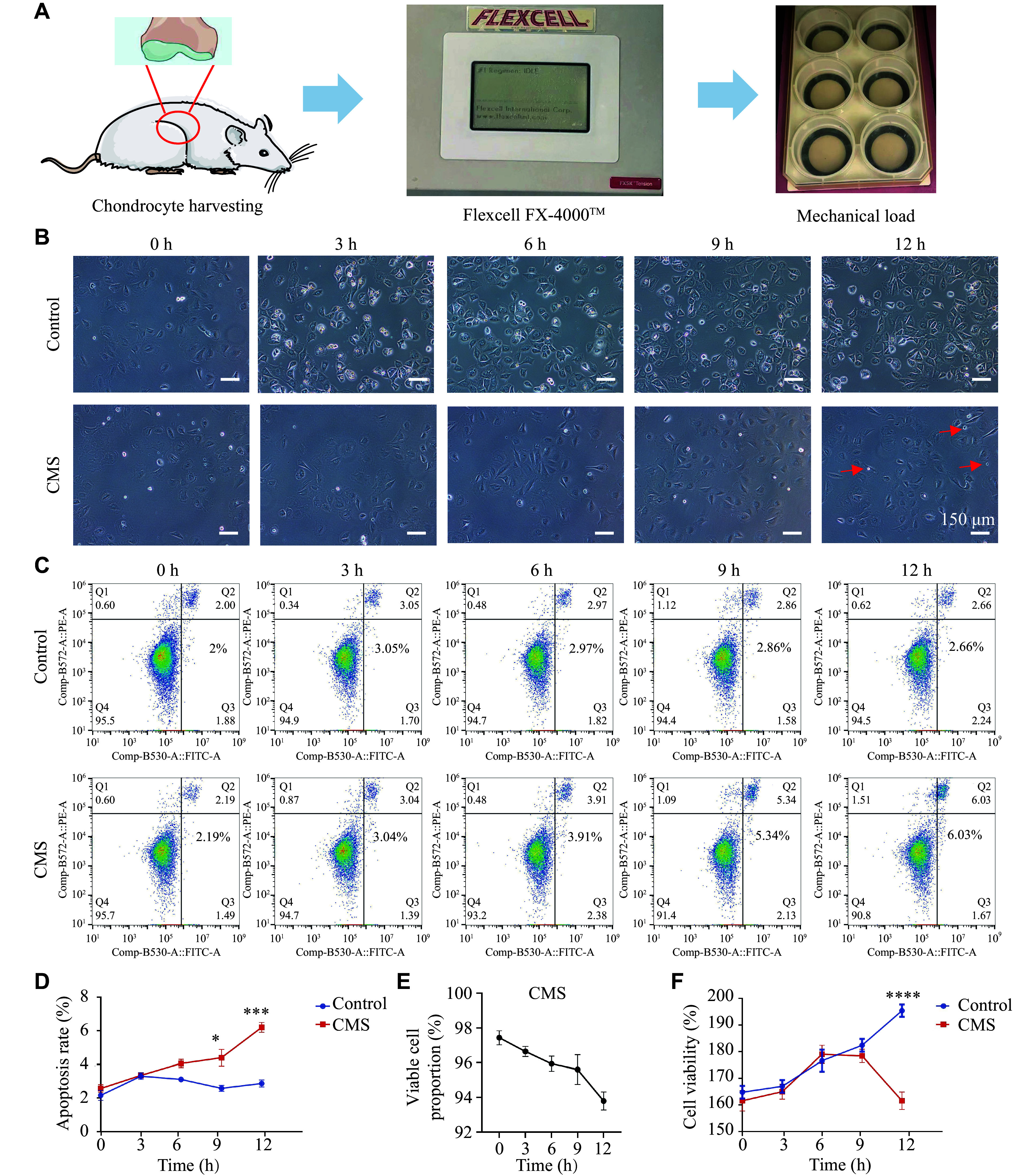
Mechanical loading induced apoptosis of chondrocytes *in vitro*. A: Illustration of mechanical loading on chondrocytes *in vitro*. B: Morphological micrographs of chondrocytes in the control and cyclic mechanical strain (CMS) groups. C and D: Apoptosis of chondrocytes after different loading durations was detected by flow cytometry. E: The proportion of viable cells in the CMS group was quantified by flow cytometry (*n* = 3). F: The cell ability of chondrocytes in the control and CMS groups was measured by CCK-8 assay (*n* = 3). Data are presented as mean ± standard deviation. Statistical analysis was performed using an unpaired Student's *t*-test. ^***^P < 0.001 and ^****^P < 0.0001.

First, we isolated and cultured rat hip joint chondrocytes. The experimental parameters, including the strength and frequency of mechanical loading, were established according to previous studies^[21–23]^. The chondrocytes in the cyclic mechanical strain (CMS) group were cultured under mechanical loading (7.5% strain, 1 Hz) and were evaluated at 0, 3, 6, 9, and 12 h after the initiation of tensile strain (***Fig. 1***). Under mechanical loading, chondrocytes remained viable and proliferated; however, the proliferation rate and cell density significantly decreased under prolonged mechanical loading. In addition, the cell morphology changed from round to polygonal, the cytoplasmic content decreased, and the cartilage nodules gradually disappeared (***Fig. 1B***). Flow cytometry analysis revealed that chondrocyte viability was maintained up to 9 h; however, at 12 h, the late apoptotic rate of chondrocytes in the CMS group was significantly higher than that in the control (NC) group (***Fig. 1C***–***1E***). These findings indicate that while mechanical loading may not lead to immediate chondrocyte apoptosis, prolonged exposure can eventually trigger apoptotic response. Additionally, the CCK-8 assay also corroborated these findings. Before 9 h, mechanical loading did not have a significant effect on cell viability. However, after 9 h, cellular viability was significantly reduced (***Fig. 1F***). These results indicate that even moderate mechanical loading, when applied over a prolonged period, can induce chondrocyte apoptosis *in vitro*.

### Long-term mechanical loading induced an inflammatory microenvironment *in vitro*

**Figure 2:**
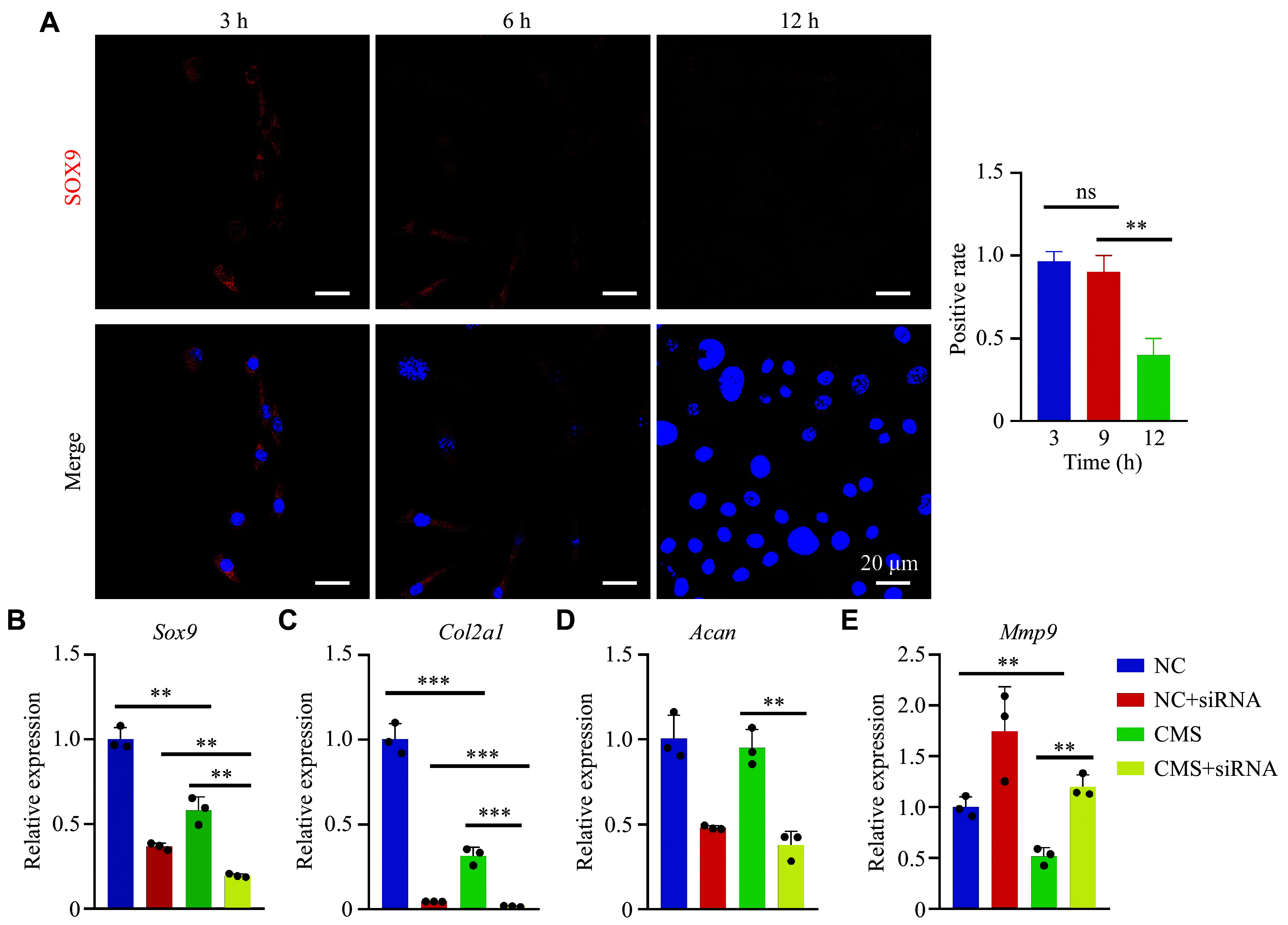
Effects of mechanical loading on expressions of chondrocyte markers and inflammatory response *in vitro*. A: Representative confocal images of immunofluorescence staining of SOX9 in chondrocytes after mechanical loading *in vitro* (*n* = 3). B–E: After mechanical loading, the mRNA expressions of *Sox9*, *Col2*, *Acan*, and *Mmp9* in chondrocytes were detected by reverse transcription-quantitative PCR (*n* = 3). Data are presented as mean ± standard deviation. Statistical analysis was performed using an unpaired Student's *t*-test for two-group comparisons and one-way ANOVA followed by Bonferroni correction for multiple-group comparisons. ^**^P < 0.01 and ^***^P < 0.001.

IF staining showed that the expression levels of SOX9, a major marker of chondrocytes, did not change significantly at 3–6 h but decreased significantly after 9 h of mechanical loading (***Fig. 2A***). This was also consistent with the current understanding that mechanical loading has a bidirectional effect on chondrocytes. Chondrocytes are key resident cells in articular cartilage and growth plates, playing a critical role in maintaining cartilage homeostasis and transducing mechanical signals. RT-qPCR analysis showed that genes associated with chondrocyte phenotype, including *Sox9* and *Col2a1*, were significantly downregulated after mechanical loading (***Fig. 2B*** and ***2C***). However, *Acan* expression did not change significantly, which may reflect the time required for the inflammatory microenvironment to suppress proteoglycan synthesis in chondrocytes (***Fig. 2D***). Under mechanical loading conditions, *Sox9* knockdown further reduced *Col2a1* expression (***Fig. 2C***), while *Acan* expression was also markedly downregulated following *Sox9* knockdown (***Fig. 2D***). Additionally, after *Sox9* silencing, *Col2a1* and *Acan* expression levels were similar in groups with or without mechanical loading (***Fig. 2B***–***2D***). Notably, *Sox9* silencing upregulated the expression of *Mmp9* both in the presence and absence of mechanical loading (***Fig. 2E***), suggesting that the mechanical loading-induced inflammatory microenvironment is closely associated with chondrocyte-dependent responses.

### Mechanical loading induced an inflammatory microenvironment and exacerbated cartilage degeneration *in vivo*

**Figure 3:**
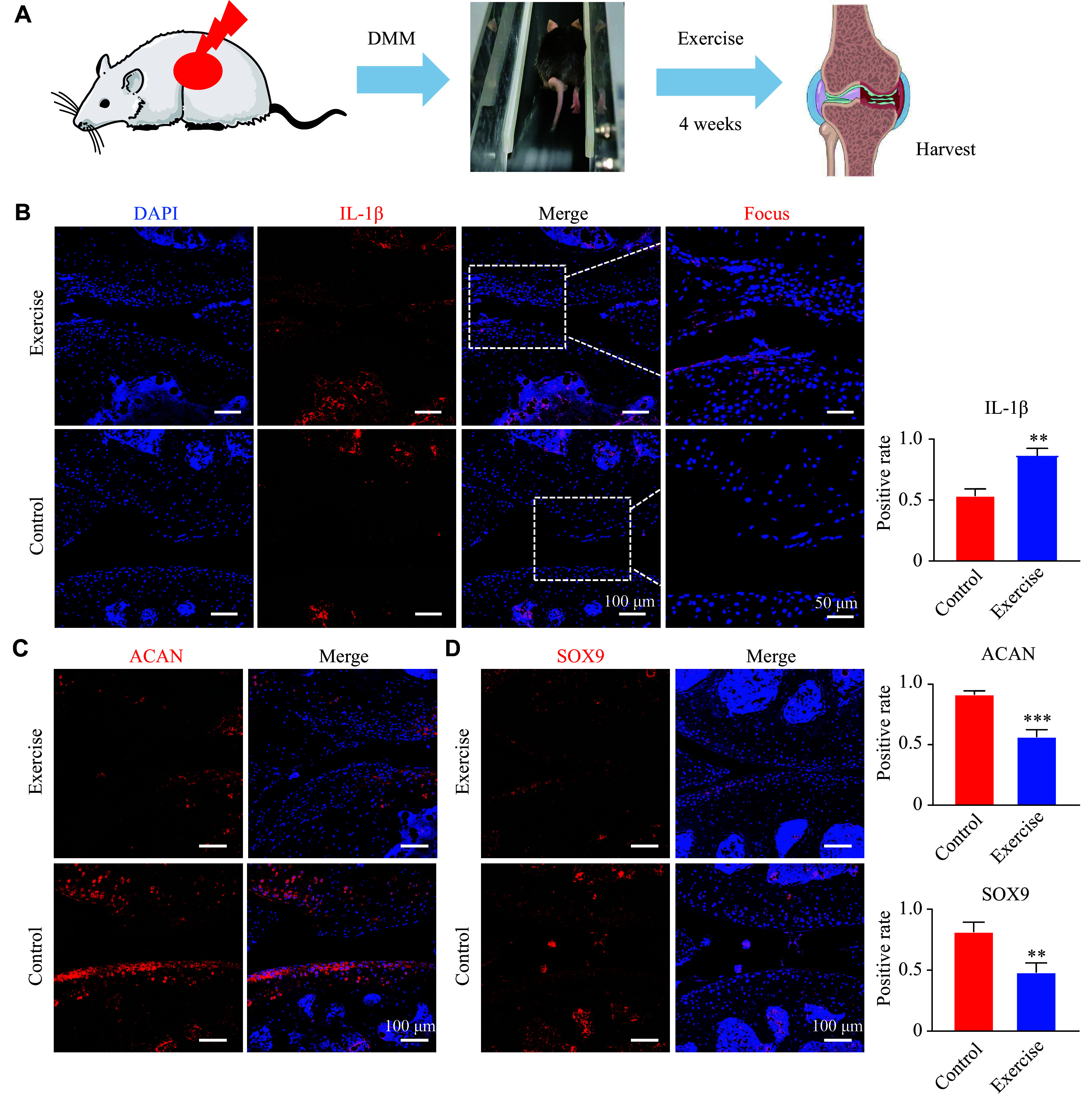
Mechanical loading induced apoptosis of chondrocytes and an inflammatory microenvironment *in vivo*. A: Illustration of exercise in mice. Mice underwent destabilization of the medial meniscus (DMM) with or without daily exercise (*n* = 5, exercise for 4 weeks). B–D: Interleukin-1β (IL-1β; B), a key inflammatory marker, along with the chondrocyte markers ACAN (C) and SOX9 (D), in the articular cartilage region were detected by immunofluorescence staining. ^**^P < 0.01 and ^***^P < 0.001 by Student's *t*-test.

To further explore whether mechanical loading in the *in vivo* environment exacerbates or alleviates early OA progression, we constructed a mouse OA model. The experimental group underwent treadmill training following OA modeling to apply mechanical loading, while the control group underwent OA modeling alone (***Fig. 3A***). Compared with the non-exercise group, the knee joint of mice in the mechanical loading group exhibited an inflammatory microenvironment. IL-1β was primarily localized in the superficial transitional layer of articular cartilage rather than in the deep calcified cartilage layer (***Fig. 3B***). Additionally, we detected chondrocyte-related markers and found that aggrecan was significantly downregulated in the articular cartilage surface, especially at the joint contact surface (***Fig. 3C***). IF staining further showed that SOX9 expression was significantly downregulated not only in the articular cartilage contact surface but also in subchondral bone (***Fig. 3D***). These findings suggest that excessive mechanical loading accelerates cartilage degeneration during the early stages of OA, primarily by promoting the loss of chondrocyte phenotype through the induction of an inflammatory microenvironment.

### Mechanical loading induced an inflammatory microenvironment and degeneration of human cartilage

**Figure 4:**
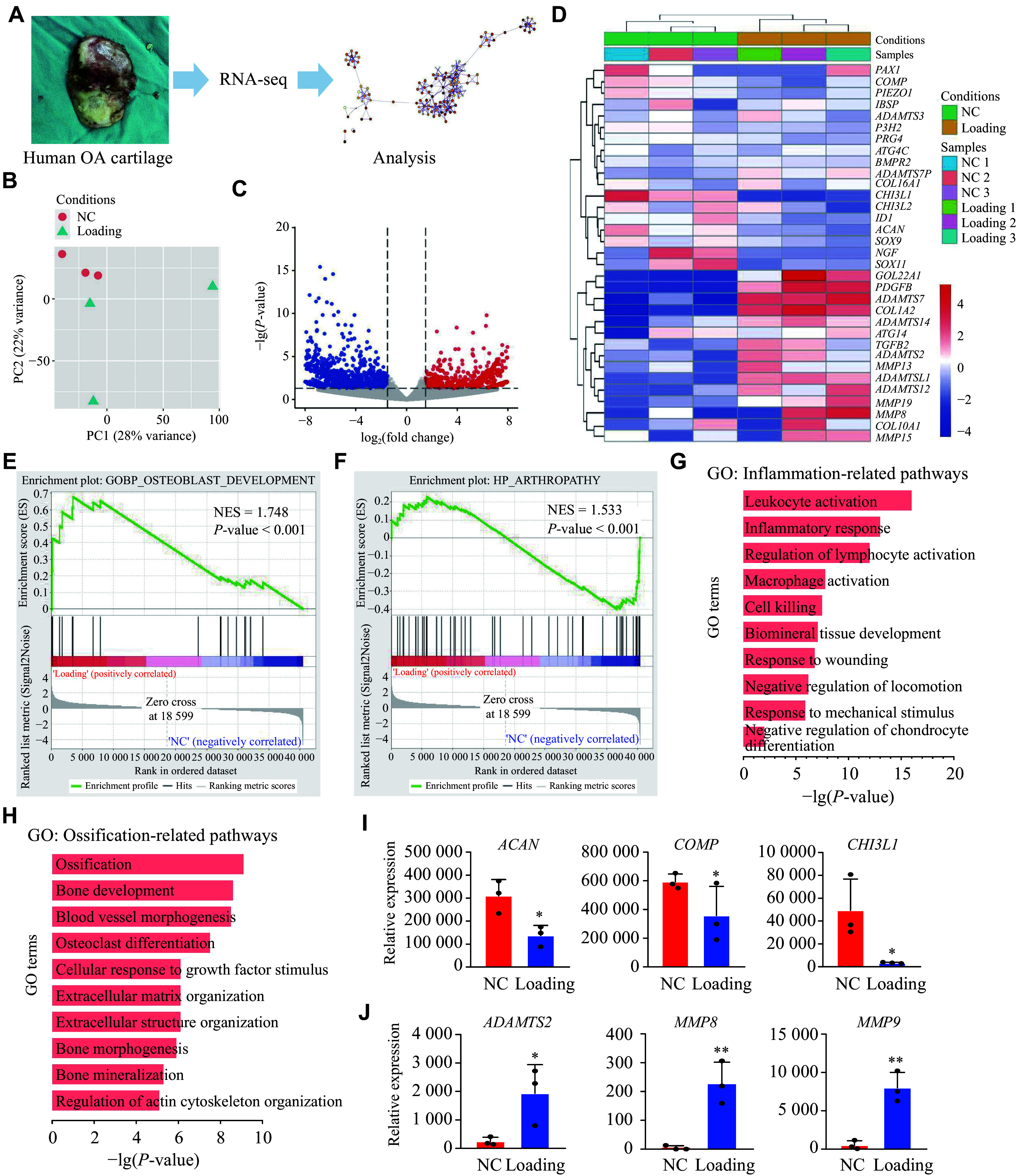
RNA-seq analysis of human articular cartilage under mechanical loading. A: Experimental workflow for human cartilage collection (*n* = 3 per group), RNA-seq, and analysis. B: Principal component analysis of transcriptomes of non-weight-bearing (NC) and weight-bearing (Loading) samples (*n* = 3). C: Volcano plot of differentially expressed genes (|log_2_FC| > 1.5, FDR < 0.05, *n* = 3). D: Heatmap of selected osteoarthritis and chondrocyte-related genes (Z-score scaled, blue = low, red = high). E and F: GSEA results showing normalized enrichment scores (NES) and FDR for selected pathways. G and H: Enriched biological processes (GO) in the mechanical loading group compared with the NC group. I: Expression of chondrocyte markers (*ACAN*, *COMP*, *CHI3L1*) from RNA-seq [log_2_(normalized counts); *n* = 3]. J: Expression of matrix-degrading genes (*ADAMTS2*, *MMP8*, and *MMP9*). Data shown as mean ± standard deviation. Two-group comparisons used unpaired Student's *t*-test. ^*^P < 0.05 and ^**^P < 0.01. Abbreviations: RNA-seq, RNA sequencing; FC, fold change; FDR, false discovery rate.

To further investigate how mechanical loading regulates the fate of chondrocytes, articular cartilage samples were obtained from both weight-bearing and non-weight-bearing regions of the same patient undergoing joint arthroplasty, thereby minimizing interindividual variability associated with age, sex, and other potential confounding factors. Bulk RNA sequencing was performed to characterize the gene expression patterns in articular cartilage (***Fig. 4A***). Principal component analysis (PCA) and heatmap analysis demonstrated that samples from the non-weight-bearing region clustered together and were clearly separated from those from the weight-bearing region (***Fig. 4B*** and ***4D***). Volcano plots showed 1230 upregulated and 883 downregulated genes in the high mechanical loading group [|log_2_(fold change)| >1.5; *P* < 0.05], compared with the low mechanical loading group (***Fig. 4C***).

### Mechanical loading triggered inflammatory and apoptotic signaling pathways

To further understand the signal mechanisms underlying cartilage degeneration induced by excessive mechanical loading, we analyzed the biological processes enriched among the differentially expressed genes (DEGs) using gene set enrichment analysis (GSEA). The results showed that the upregulated genes in the weight-bearing group were enriched in pathways related to osteoclast development and arthritis-associated signaling (***Fig. 4E*** and ***4F***). GO enrichment pathway analysis further revealed significant enrichment in pathways related to inflammatory activation (*e.g*., leukocyte activation, macrophage activation, and inflammatory response) and aberrant ossification-related signaling pathways (*e.g*., ossification, osteoclast differentiation, bone mineralization, and extracellular matrix organization) (***Fig. 4G*** and ***4H***). Additionally, chondrocyte-related genes, including *COMP*, *CHI3L1*, and *ACAN*, were significantly downregulated, while genes related to inflammation and apoptosis, such as *MMP9*, *ADAMTS2*, and *MMP8*, were significantly upregulated in the weight-bearing (Loading) group, compared with the non-weight-bearing (NC) group (***Fig. 4I*** and ***4J***). These findings indicate that excessive mechanical loading induces a characteristic inflammatory microenvironment in the cartilage at the transcriptional level, suggesting that high-intensity or prolonged mechanical loading may contribute to OA progression rather than promote cartilage recovery.

### Excessive mechanical loading upregulated neural NGF signal expression

**Figure 5:**
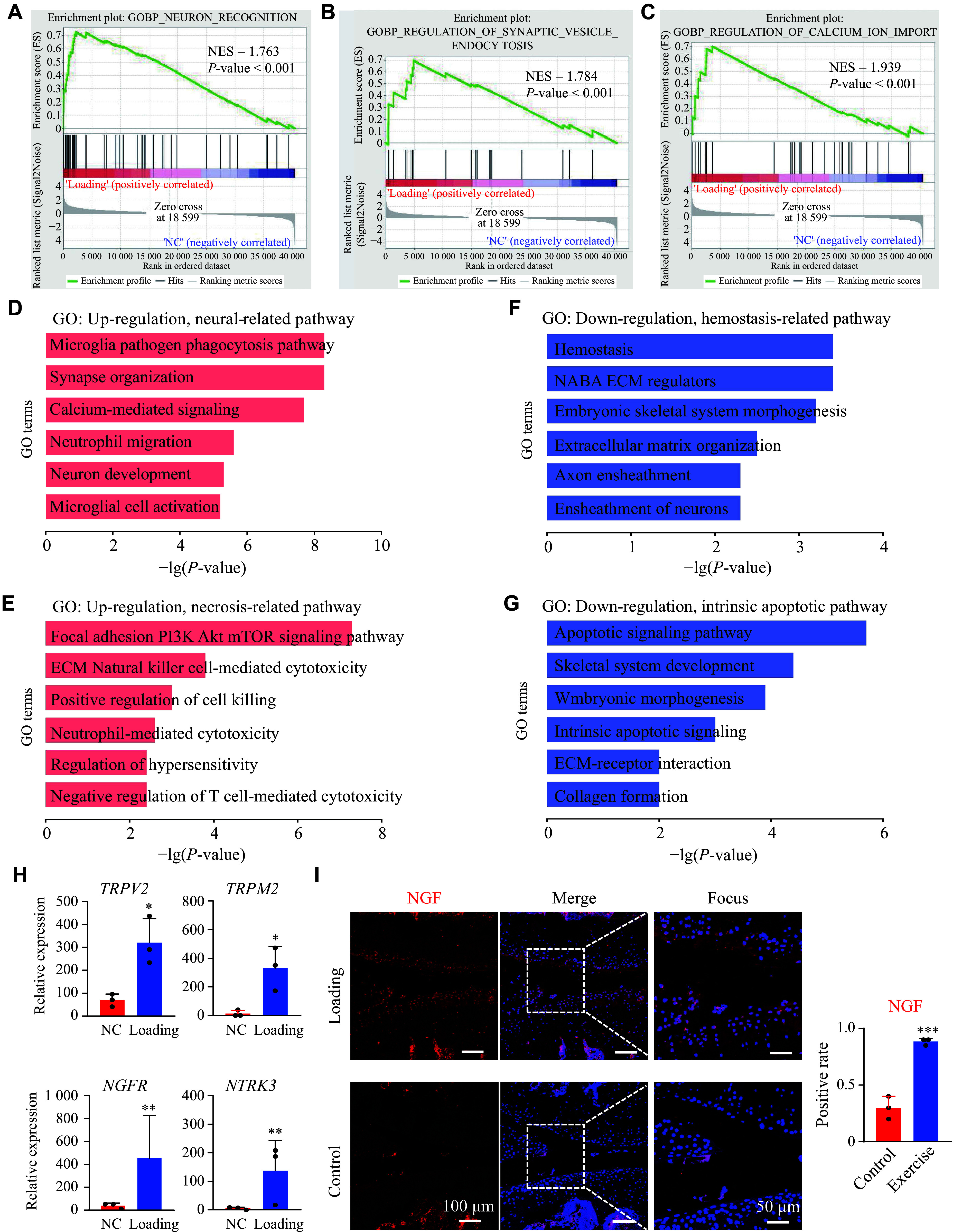
Excessive mechanical loading enriched neural-related pathways and induced an increase in nerve growth factor (NGF) signals. A–C: Gene Set Enrichment Analysis (GSEA) of neuron recognition, synaptic vesicle endocytosis, and regulation of calcium ion import. D and E: Enriched biological processes (GO) for upregulated genes in the weight-bearing (loading) group compared with the non-weight-bearing (NC) group, including neural-related pathways and necrosis-related pathways. F and G: Enriched biological processes (GO) for downregulated genes in the loading group compared with the NC group, including hemostasis-related pathways and intrinsic apoptotic pathways. H: Expression of neuro-related genes (*TRPV2*, *TRPM2*, *NGFR*, *NTRK3*) from RNA-seq data [log_2_(normalized counts relative to library size); *n* = 3 per group, unpaired *t*-test]. Data are presented as mean ± standard deviation. I: Immunofluorescence staining of NGF in articular cartilage after mechanical loading in mice. ^*^P < 0.05 and ^**^P < 0.01. Abbreviations: NES, normalized enrichment score; FDR, false discovery rate.

We further investigated the mechanism of mechanical loading-induced inflammatory microenvironment beyond the ossification phenotype observed in the previous section. GSEA revealed significant enrichment in pathways related to neuronal recognition, synaptic vesicle endocytosis, and regulation of calcium ion import (***Fig. 5A***–***5C***). GO analysis of neuronal pathways also revealed significant enrichment for upregulated genes in pathways related to nerve and microglia activation (*e.g.*, microglia pathogen phagocytosis, synapse organization, neutrophil migration, and calcium-mediated signaling) as well as downstream pathways associated with autophagy and cytotoxicity (*e.g.*, focal adhesion PI3K-Akt-mTOR signaling pathway, neutrophil-mediated cytotoxicity, ECM natural killer cell-mediated cytotoxicity, positive regulation of cell killing, and negative regulation of T cell-mediated cytotoxicity) (***Fig. 5D*** and ***5E***), while downregulated genes were enriched in pathways related to ECM homeostasis and physiological apoptosis, including hemostasis, extracellular matrix organization, intrinsic apoptotic signaling, ECM-receptor interaction, and collagen formation (***Fig. 5F*** and ***5G***).

We further compared the changes in specific genes related to neural signaling, and found that *TRPV2* and *TRPM2*, both of which are major channels involved in calcium ion signal transduction and important mediators of nerve electrophysiological signal coupling, were significantly upregulated in the high mechanical loading group. We also observed significant upregulation of *NGFR* and *NTRK3* expression in the high mechanical loading group (***Fig. 5H***). Both genes encode receptors with nerve growth-stimulating activity and are involved in regulating the growth and differentiation of sympathetic neurons and sensory neurons. IF staining showed that NGF expression was confined to the bone marrow region in the control group (***Fig. 5I***). However, NGF expression was significantly elevated in the articular cartilage of the mechanically loaded OA model, suggesting that neural signaling is closely involved in the OA process.

## Discussion

In patients with knee or hip OA undergoing non-surgical treatment, there is a significant correlation between mechanical loading and disease severity, which is characterized by increased pain levels and impaired motor function^[24–26]^. In clinical practice, mechanical loading represents a double-edged sword in the management of OA symptoms^[27]^. Clinical practice guidelines from the American Academy of Orthopaedic Surgeons recommend exercise therapy and weight management as primary approaches for hip or knee OA, aiming to optimize mechanical loading and alleviate joint symptoms^[28–29]^. However, in real-world clinical practice, exercise does not always yield the expected outcomes. For example, high-impact activities such as repetitive jumping and running have been reported to place substantial mechanical stress on the joints, thereby accelerating OA progression^[30–32]^. In the current study, our objective was to investigate whether long-term, lower-intensity mechanical loading might provide protective effects on articular cartilage.

There is increasing evidence that mechanical loading plays a pivotal role in OA development, exerting either beneficial or detrimental effects depending on its intensity, frequency, and mode^[33–35]^. In the current study, we constructed an *in vitro* mechanical loading model to assess the effects of sustained mechanical stimulation on chondrocytes. Initially, no significant changes were observed in chondrocyte apoptosis after early exposure to mechanical stimulation. However, over time, chondrocytes exhibited reduced proliferative capacity, decreased cell density, and loss of their typical rounded and translucent morphology. This suggests that even mild, long-term mechanical loading contributes to OA progression by inducing chondrocyte apoptosis. Moreover, prolonged mechanical stress generated a localized pro-inflammatory microenvironment, as indicated by the upregulation of IL-1β and the downregulation of key chondrogenic markers, including *Sox9*, *Acan*, and *Col2a1* *in vitro*.

We further established a treadmill-based animal model to mimic long-term mechanical loading. This model revealed that the inflammatory microenvironment primarily affected the superficial transitional layer of articular cartilage. A significant downregulation of cartilage-related markers was observed in this layer, suggesting that it is the primary site of biological responses to mechanical loading. Importantly, our findings suggest that even moderate-intensity exercise, when prolonged, may result in cartilage damage through NGF-mediated pathways. We therefore propose that individuals with early-stage OA may benefit from limiting high-impact or prolonged exercise sessions, for example, to less than 30 min per session. This provides stage-specific guidance for exercise therapy in OA and aligns with current rehabilitation guidelines.

Our findings also emphasize the critical role of load duration, rather than load magnitude alone, in OA pathology. Prolonged mechanical loading (> 12 h) at approximately 7.5–10% strain aggravated chondrocyte apoptosis and inflammation, supporting clinical recommendations for low-impact exercises (*e.g.*, cycling) in early-stage OA. RNA-seq analysis further revealed that sustained loading activated nerve-sensitization pathways, particularly NGF/TrkA signaling, suggesting that intermittent exercise protocols (*e.g.*, 30-minute sessions with rest intervals) may reduce cumulative cartilage damage. These findings provide a mechanistic basis for optimizing exercise prescriptions in OA rehabilitation according to disease stage.

To further explore the mechanisms linking mechanical loading and OA progression, we conducted RNA-seq analysis of cartilage samples from animals subjected to prolonged weight-bearing. This analysis identified enrichment of pathways related not only to ossification, osteoblast activity, and inflammation, but also to neurogenic sensitization, particularly involving NGF signaling. We validated these transcriptomic findings *in vivo* and demonstrated NGF upregulation in mechanically loaded cartilage. These results suggest a potential feedforward mechanism in which mechanical loading promotes NGF expression, which in turn exacerbates chondrocyte inflammation and apoptosis, thereby accelerating OA progression. This mechanistic insight helps reconcile NGF's dual role in pain sensitization and structural degradation.

In light of the emerging therapeutic relevance of NGF, we also highlight its potential role as a biomarker for monitoring joint responses to mechanical loading and exercise. NGF expression levels could serve as a guide for tailoring exercise intensity and duration, thereby facilitating personalized rehabilitation strategies for OA patients.

Nonetheless, our study has several limitations. First, although *in vitro* and animal models provided mechanistic insights into load-induced chondrocyte dysfunction, these models cannot fully replicate the complex biomechanics, neuromuscular feedback, and systemic interaction present in human joints. Second, the *in vitro* environment excludes systemic biological factors, such as endocrine and metabolic influences. Finally, although our results are consistent with clinical observations linking high-impact activity to OA progression, longitudinal clinical studies directly correlating exercise parameters with NGF levels and cartilage outcomes are still necessary. Future research should integrate biomechanical monitoring and NGF profiling in OA patients to validate and refine these findings.

In conclusion, we employed both *in vivo* and *in vitro* mechanical loading models to explore the impact of prolonged mechanical stress on OA. Our results demonstrated that prolonged mechanical loading induces chondrocyte apoptosis, promotes a pro-inflammatory microenvironment in the knee joint, and accelerates cartilage degeneration. Transcriptomic analysis of human cartilage further revealed a significant enrichment of neurogenic signaling pathways, particularly those involving NGF, in cartilage exposed to sustained mechanical stress.

These findings suggest that even moderate-intensity exercise, when performed over extended durations, may exacerbate NGF-mediated cartilage damage. Based on the NGF upregulation observed in our study, we recommend that individuals with early-stage OA limit high-impact physical activity to less than 30 min per session. Moreover, NGF may serve as a promising biomarker for assessing joint tolerance to mechanical loading, offering a potential tool to guide personalized exercise prescriptions in OA rehabilitation.

Collectively, our work provides mechanistic insight into how mechanical loading contributes to OA pathogenesis and offers actionable recommendations to inform exercise guidelines for at-risk populations.

## References

[1] (2020). Osteoarthritis in 2020 and beyond: A *Lancet* Commission. Lancet.

[2] (2020). 2019 American College of Rheumatology/Arthritis Foundation guideline for the management of osteoarthritis of the hand, hip, and knee. Arthritis Care Res (Hoboken).

[3] (2021). Subchondral physiology and vasculo-mechanical factors in load transmission and osteoarthritis. Bone Joint Res.

[4] (2021). Association between changes in knee load and effusion-synovitis: Evidence of mechano-inflammation in knee osteoarthritis using high tibial osteotomy as a model. Osteoarthritis Cartilage.

[5] (2024). Enhanced surface immunomodification of engineered hydrogel materials through chondrocyte modulation for the treatment of osteoarthritis. Coatings.

[6] (2021). Osteocyte dysfunction in joint homeostasis and osteoarthritis. Int J Mol Sci.

[7] (2024). Pathogenic mechanisms and therapeutic approaches in obesity-related knee osteoarthritis. Biomedicines.

[8] (2021). Mechanotransducive biomimetic systems for chondrogenic differentiation *in vitro*. Int J Mol Sci.

[9] (2024). Unveiling the role of hypertrophic chondrocytes in abnormal cartilage calcification: Insights into osteoarthritis mechanisms. Eur Cells Mater.

[10] (2022). Mechanical unloading of engineered human meniscus models under simulated microgravity: A transcriptomic study. Sci Data.

[11] (2022). Exercise-induced piezoelectric stimulation for cartilage regeneration in rabbits. Sci Transl Med.

[12] (2016). Subchondral plate porosity colocalizes with the point of mechanical load during ambulation in a rat knee model of post-traumatic osteoarthritis. Osteoarthritis Cartilage.

[13] (2024). A damaging *COL6A3* variant alters the *MIR31HG*-regulated response of chondrocytes in neocartilage organoids to hyperphysiologic mechanical loading. Adv Sci (Weinh).

[14] (2021). Obesity and load-induced posttraumatic osteoarthritis in the absence of fracture or surgical trauma. J Orthop Res.

[15] (2023). Inflammaging and osteoarthritis. Clin Rev Allergy Immunol.

[16] (2022). Obesity, inflammation, and immune system in osteoarthritis. Front Immunol.

[17] (2019). Effect of tanezumab on joint pain, physical function, and patient global assessment of osteoarthritis among patients with osteoarthritis of the hip or knee: A randomized clinical trial. JAMA.

[18] (2024). Britanin alleviates chondrocyte ferroptosis in osteoarthritis by regulating the Nrf2-GPX4 axis. Arab J Chem.

[19] (2019). Mechanoflammation in osteoarthritis pathogenesis. Semin Arthritis Rheum.

[20] (2019). Therapeutic options for targeting inflammatory osteoarthritis pain. Nat Rev Rheumatol.

[21] (2024). Moderate mechanical stress suppresses chondrocyte ferroptosis in osteoarthritis by regulating NF-κB p65/GPX4 signaling pathway. Sci Rep.

[22] (2021). Moderate mechanical stress suppresses the IL-1β-induced chondrocyte apoptosis by regulating mitochondrial dynamics. J Cell Physiol.

[23] (2023). Mechanical stress protects against chondrocyte pyroptosis through TGF-β1-mediated activation of Smad2/3 and inhibition of the NF-κB signaling pathway in an osteoarthritis model. Biomed Pharmacother.

[24] (2025). Cartilage deformation, outcomes, and running force comparisons in females with and without knee injuries. J Sport Rehabil.

[25] (2025). Correlation between various loads and apoptosis in medial platform chondrocytes in knee varus deformity. Clin Biomech (Bristol).

[26] (2025). Effects of kinematic and kinetic variables on articular cartilage mechanical and biological properties. Osteoarthritis Cartilage.

[27] (2025). Role and mechanism of mechanical load in the homeostasis of the subchondral bone in knee osteoarthritis: A comprehensive review. J Inflamm Res.

[28] (2025). 2024 American Academy of Orthopaedic Surgeons (AAOS) clinical practice guideline: Management of osteoarthritis of the hip (summary) interpretation. Chin J Rep Reconstruct Surg.

[29] (2025). Decreasing trends in arthroscopic treatment of knee osteoarthritis after publication of the 2013 Academy of Orthopaedic Surgeons Clinical Practice Guidelines. J Am Acad Orthop Surg Glob Res Rev.

[30] (2025). Effects of training modalities and additional pain education on exercise-induced hypoalgesia in people with osteoarthritis of the knee: A randomised controlled feasibility trial. Eur J Pain.

[31] (2025). Exercise-related changes in knee articular structures detected using magnetic resonance imaging T1ρ and T2 mapping. Eur J Radiol Open.

[32] (2025). Novel three-dimensional preclinical model for investigating cartilage regeneration, incorporating physiological and pathological mechanical loading. Tissue Eng Part C: Methods.

[b33] 33Shen Y, Sun W, Liu Y, et al. Mechanical stress promotes synovial inflammation and osteoarthritis development via the NF-κB p52/IL-6 signaling pathway[J]. Rheumatology, 2025, doi: 10.1093/rheumatology/keaf553. [Epub ahead of print].

[34] (2025). Mechanical stimulation in 2D: A potent accelerator of matrix mineralization in ATDC5 chondrogenic cells. J Orthop.

[35] (2025). The post-operative loading regimen influences the regenerative potential of a biomimetic osteochondral scaffold. Biomed Eng Online.

